# Soluble Serum CD81 Is Elevated in Patients with Chronic Hepatitis C and Correlates with Alanine Aminotransferase Serum Activity

**DOI:** 10.1371/journal.pone.0030796

**Published:** 2012-02-15

**Authors:** Martin-Walter Welker, David Reichert, Simone Susser, Christoph Sarrazin, Yolanda Martinez, Eva Herrmann, Stefan Zeuzem, Albrecht Piiper, Bernd Kronenberger

**Affiliations:** 1 Medizinische Klinik 1, Klinikum der Johann Wolfgang Goethe-Universität, Theodor-Stern-Kai 7, Frankfurt am Main, Germany; 2 Fachbereich Medizin, Institut für Biostatistik und mathematische Modellierung, Johann Wolfang Goethe-Universität, Frankfurt am Main, Germany; Karolinska Institutet, Sweden

## Abstract

**Aim:**

Cellular CD81 is a well characterized hepatitis C virus (HCV) entry factor, while the relevance of soluble exosomal CD81 in HCV pathogenesis is poorly defined. We performed a case-control study to investigate whether soluble CD81 in the exosomal serum fraction is associated with HCV replication and inflammatory activity.

**Patients and Methods:**

Four cohorts were investigated, patients with chronic hepatitis C (n = 37), patients with chronic HCV infection and persistently normal ALT levels (n = 24), patients with long term sustained virologic response (SVR, n = 7), and healthy volunteers (n = 23). Concentration of soluble CD81 was assessed semi-quantitatively after differential centrifugation ranging from 200 g to 100,000 g in the fifth centrifugation fraction by immunoblotting and densitometry.

**Results:**

Soluble CD81 was increased in patients with chronic hepatitis C compared to healthy subjects (p = 0.03) and cured patients (p = 0.017). Patients with chronic HCV infection and persistently normal ALT levels and patients with long term SVR had similar soluble CD81 levels as healthy controls (p>0.2). Overall, soluble CD81 levels were associated with ALT levels (r = 0.334, p = 0.016) and severe liver fibrosis (p = 0.027).

**Conclusion:**

CD81 is increased in the exosomal serum fraction in patients with chronic hepatitis C and appears to be associated with inflammatory activity and severity of fibrosis.

## Introduction

Chronic hepatitis C virus (HCV) infection is a major cause of liver cirrhosis, and hepatocellular carcinoma worldwide [1], [2]. Recently the protease inhibitors boceprevir and telaprevir, which are associated with considerably enhanced sustained virologic response (SVR) rates in HCV genotype 1 infected patients in combination with pegylated interferon-alfa and ribavirin, have been approved by the European Medicines Agency and the Food and Drug Administration [3]–[5]. Nevertheless, approximately one quarter of patients with HCV genotype 1 infection are assumed not to achieve SVR even with triple therapy including either boceprevir or telaprevir [6].

HCV is a plus-strand RNA virus without known ability to integrate its genome into the host genome. Chronic hepatitis C is characterized by high turnover of infected cells and persistence of HCV requires continuous *de novo* infection [7]–[10]. Therefore, inhibition of cell entry is a promising approach in antiviral therapy against HCV. Moreover, cell entry inhibitors may be useful in prevention of otherwise inevitable graft infection after orthotopic liver transplantation in HCV associated liver cirrhosis and hepatocellular carcinoma. Proof-of-concept studies suggest that the new class of HCV entry inhibitors has a substantial capability to widen preventive and therapeutic options in HCV infection [11].

Four essential HCV hepatocyte entry receptors, CD81, the scavenger receptor B1, claudin-1, and occludin have been identified, yet [12]–[15]. CD81 is of particular interest because CD81 is not only expressed on hepatocytes but also on peripheral blood mononuclear cells (PBMC). Interaction of HCV with CD81 expressed on PBMC was suggested to be involved in an attenuated innate and adaptive immune response against HCV as well as in development of extrahepatic HCV manifestations [16]–[20]. However, these results were challenged by a recent study showing that HCV envelope 2 proteins have no modulatory effect on natural killer cell functions when expressed as part of cell culture infectious viral particles [21]. The different observations could be explained by differences in the configuration of viral envelope proteins in protein expression systems compared to systems using compete HCV particles [20], [21].

Yet well known as a cell surface protein, CD81 is also a typical component of exosomes. Exosomes are membrane vesicles secreted by different eukaryotic cells, e.g. hepatocytes and lymphocytes [22]–[24]. In two studies, exosomes were enriched by differential centrifugation from human plasma from patients with chronic hepatitis C [23], [25]. Exosomal CD81 was reliably detectable in the pellet of the fifth fraction of the centrifugation process. Moreover, CD81 exosomes were found to be associated with HCV RNA in plasma obtained from patients chronically infected with HCV [25]. However, neither CD81 levels nor HCV RNA were quantified in enriched plasma or serum, which makes it difficult to assess clinical importance of these findings. Nevertheless, exosomal CD81 may be of relevance in chronic HCV infection as HCV particles bound to exosomal CD81 could represent an additional HCV compartment with putative impact on the HCV infection rate or viral persistence, respectively [8].

Aim of the present study was to perform a comprehensive analysis if soluble CD81 is increased in patients with chronic hepatitis C compared to healthy controls, patients with chronic HCV infection but persistently normal alanine transaminase (ALT) levels, and patients with cured hepatitis C. Therefore, a method to quantify soluble CD81 in the exosomal serum fraction was established, and the association of soluble CD81 in the exosomal serum fraction with clinical and virological parameters within the different cohorts was investigated.

## Methods

### Patients

The present study is a case control study with 23 healthy controls and 37 patients with chronic hepatitis C and elevated alanine aminotransferase (ALT) level measured at least at 2 time points 14 days or more apart within 6 months before study entry. Furthermore, 24 patients with chronic HCV infection and persistently normal ALT serum levels as documented by three occasional measurements within the last 6 months before study entry and a minimum interval of 4 weeks between measurements, and 7 patients with long-term sustained virologic response 5 years after antiviral therapy with pegylated interferon-alfa 2a and ribavirin were included. All cohorts were independently recruited. Patients with decompensated liver cirrhosis or co-infection with hepatitis B virus or human immunodeficiency virus were excluded. Diagnosis of chronic HCV infection (>6 months) was confirmed by positive anti-HCV antibodies by third-generation enzyme immunoassay and detectable HCV RNA in serum. Grading and staging of chronic hepatitis C was done by a local, experienced pathologist according to the histological activity index described by Knodell et al. [26]. Patient cohorts with persistently normal ALT levels and patients with elevated ALT levels were derived from clinical trials performed at the university hospitals Frankfurt/Main, Germany and Homburg/Saar, Germany. Study protocols were conform to the ethical guidelines of the 1975 Declaration of Helsinki and were approved by the ethic committees of the university hospitals Frankfurt/M, Germany and Homburg/Saar, Germany. Written, informed consent was obtained from every human subject when the samples were collected under the terms of study protocol.

### Isolation of CD81 from serum and plasma

Serum and plasma for ALT and AST levels, CD81 assessment, and HCV-RNA quantification were collected at the same time point and stored at −80°C until use. Enrichment of exosomes was performed according to Masciopinto et al. [25]. In this former study, the CD81 enriched exosomal fraction was identified by immunoblotting in the pellets from the fourth (p4) and - mainly - the fifth (p5) centrifugation fraction from plasma [25]. In the current study, differential centrifugation was performed at 200 g for 10 min (p1), at 500 g (p2), at 2000 g for 15 min (p3), at 10,000 g for 30 min (p4), and finally 100,000 g for 30 min (p5). The corresponding pellets (p1, p2, etc.) were each resuspended in 50 µl lysis buffer (100 mL 0.5 M TrisHCl pH 7.4, 1.5 mM NaCl, 2.5% desoxycholic acid, 10% NP-40, 10 mM ethylenediaminetetraacetic acid, diluted 1∶10 in aqua bidest., Merck, Darmstadt, Germany, and Sigma-Aldrich, Taufkirchen, Germany) and stored at −20°C until further analysis. As usage of serum may have some advantages over plasma, e.g. better reflection of biological conditions and easier sample processing, we further optimized detection of CD81 in enriched serum. Therefore, we first compared isolation of not cell-bound CD81 from plasma and serum samples, and then tested different volumes of serum (30 mL, 15 mL, 7 mL, and 1 mL) to evaluate a possible minimum amount for reliable detection of CD81. In contrast to plasma, serum samples were allowed to clot for 10 min before centrifugation. After that, serum specimen of the healthy controls and distinct patient groups were processed using the developed optimized serum sample processing protocol.

### Immunoblotting of CD81

Serum derived proteins from different centrifugation steps were separated by dodecylsulfate polyacrylamide gel electrophoresis (SDS-PAGE). As thiol compounds may have a negative impact on anti-CD81 antibody binding [27], a thiol free SDS sample buffer was used. In detail, after dilution of specimens in loading buffer (5 mL glycerin, 2 mL 0.625 M TrisHCl, pH 6,8, 0.2 g SDS, 0.1 mL bromphenoleblue as 1% ethanol dilution, 2.4 mL aqua bidest.), the samples were separated by SDS-page (50 mA, 90 min) and electrophoretic transferred (110 mA, 90 min) to nitrocellulose membranes Whatman Schleicher & Schell, Dassel, Germany). Nonspecific binding sites were blocked by incubation in 5% skim milk in buffer TBST (150 mM NaCl; 30 mM Tris, pH 7.4; 0.05% Tween20) for 12 hours at +4°C. Primary anti-CD81 antibody (mouse-anti-human antibody, clone JS-81, Pharmingen, Heidelberg, Germany) were allowed to bind for a minimum of 2 hours. Detection of CD81/antibody immune complexes was performed using horse radish peroxidase-conjugated antibodies (Bio-Rad Laboratories, Hercules CA, U.S.A.) and enhanced chemiluminescence (Millipore GmbH, Schwalbach, Germany). By staining with monoclonal anti-CD81 antibodies and consecutive polyclonal horse raddish peroxidase-conjugated antibodies or staining with polyclonal horse raddish peroxidase-conjugated antibodies only, specific detection of CD81 was confirmed (data not shown). Further semi-quantitative analysis was performed by automated densitometry.

### Quantification of CD81

Huh7 cells are characterized by high CD81 expression [28]. Serial dilutions of a cell lysate of 10^4^ Huh7 were used as standard for semi-quantitative assessment of serum CD81. Aliquots of the lysate were stored at −80°C until use, and applied as the same standard for all experiments. CD81 in Huh7 cell lysates was detected by immunoblotting and densitometric analysis by use of an analysis program (Gelscan 5, BioSciTec GmbH, Frankfurt, Germany). Concentration of serum derived CD81 was assessed semi-quantitatively in relation to the standard curve obtained from the Huh7 cell lysate dilutions by densitometry and expressed as relative units (RU).

### Measurement of HCV RNA from native serum and HCV genotyping

Quantitative assessment of HCV RNA in serum was performed by a quantitative reverse transcription polymerase chain reaction assay (Cobas Amplicor HCV MonitorTM 2.0, Roche Diagnostic Systems, Branchburg, NJ; lower detection limit, 600 IU/mL). Samples with HCV RNA concentration above the upper detection limit above 800.000 IU/mL were diluted appropriately. HCV genotyping was performed by reverse hybridization assay (Inno LiPA HCV II, Innogenetics, Gent, Belgium). All contamination prevention measures suggested by Kwok and Higuchi were strictly applied [29].

### Measurement of HCV RNA in enriched serum

For comparison with serum HCV RNA and correlation to CD81 concentration within enriched serum fraction p5, HCV RNA was assessed in enriched serum, also. Therefore, differential centrifugation was performed in accordance to the CD81 enrichment protocol. Each pellet from a given centrifugation step was resuspended in 0.25 mL PBS, and HCV RNA was extracted fully automatic (COBAS-Ampliprep, Roche, Mannheim, Germany). Quantification of HCV RNA was performed standardized by real-time polymerase chain reaction (COBAS-Taqman 48 Analyzer Roche, Mannheim, Germany).

### Statistical analyses

Unless indicated otherwise, all tests were two tailed and *p* values<0.05 were considered significant. Clinical and biochemical characteristics of patients were expressed as mean ± standard deviation (SD) or median and range as appropriate. Correlations between two variables were calculated by the spearman test. Furthermore, Kruskal-Wallis test, Friedman test, Wilcoxon-paired sample test and Wilcoxon Mann-Whitney U test were applied as appropriate. A Bonferroni correction was applied, if multiple comparisons were performed. A receiver operating characteristic analysis was performed to compare CD81-p5 and severity of liver fibrosis.

## Results

### Detection of CD81 in the exosomal serum fraction

**Figure 1:**
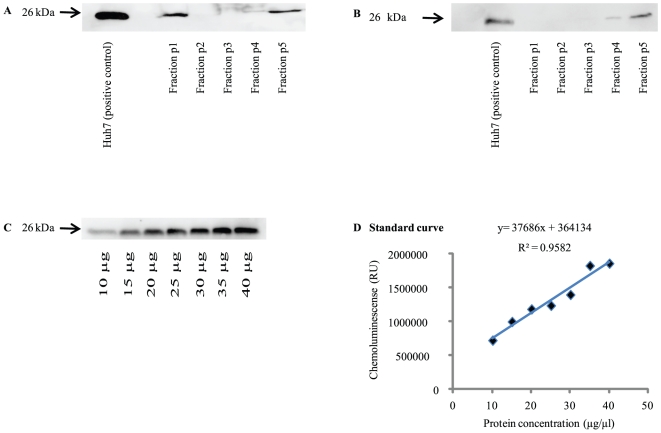
Enrichment and quantification of soluble CD81. (A, B) Immunoblotting of soluble CD81 is shown after enrichment by differential centrifugation of frozen (A) plasma and (B) serum samples. Huh7 cells served as positive control, and HepG2 cells as negative control. While only a marginal fraction was detectable in fraction p4, the main portion was detectable in fraction p5, comparable between plasma and serum samples. (C, D). Soluble CD81 concentration was assessed semi-quantitatively by use of dilutions from a Huh7 cell lysate by densitometric analysis. The lysate dilution immunoblot analysis and the corresponding obtained standard curve are shown in (C) and (D), respectively.

Detection of exosomal CD81 in plasma from patients with chronic hepatitis C has been previously described by Masciopinto et al. [25]. In the present study, we investigated whether exosomal CD81 is also detectable in human serum. For this purpose, differential centrifugation for enrichment of exosomal CD81 was performed with human plasma and serum as previously described for plasma, followed by immunoblotting with CD81 specific monoclonal antibodies [25]. Lysates from Huh7 cells characterized by high CD81 expression were used as positive control. Consistently, CD81 was absent in fractions p2 and p3, weakly detectable in fraction p4 and reliably detectable in the exosomal fraction p5 of both plasma and serum samples (Figure 1 A, B). The only difference in detection of CD81 in serum vs. plasma samples was found in the p1 fraction corresponding to the cellular fraction. Here, CD81 was detectable in plasma samples only. Fraction p5 containing exosomal CD81 was used for subsequent analyses. Enrichment of CD81 from different volumes of serum ranging from 1 mL to 30 mL showed that robust detection of CD81 was feasible with the smallest tested volume of 1 mL serum. This volume was used in all further analyses.

### Quantification of serum CD81

**Figure 2:**
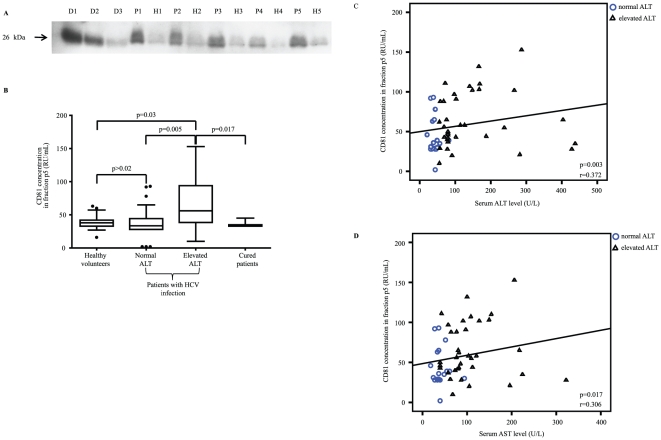
Quantification of soluble CD81 serum levels in different populations and correlation to serum ALT and AST levels. (A) Immunoblot analysis from representative patient samples (P1–P5) and healthy controls (H1–H5) as well as Huh7 dilution series (D1–D3). Enrichment of soluble CD81 had been standardized before sample processing, and a standardized volume of 1 mL serum was used for CD81 enrichment and quantification in all study samples analyzed. (B) Enriched CD81 serum concentrations given in healthy volunteers (n = 23), patients with chronic HCV infection and persistently normal ALT levels (n = 24), patients with chronic hepatitis C and elevated ALT levels (n = 37), and patients with long-term SVR (n = 7). The CD81 concentration is given in relative units (RU)/mL as box-and-whisker plot showing median, 25 and 75 quartiles as well as the total range. Differences between two groups were calculated by the Mann-Whitney U test. (C) Correlation of CD81 concentration in fraction p5 given in RU/mL and serum ALT level given in U/mL in all patients with chronic HCV infection, including patients with elevated (▵) and persistently normal (○) ALT levels (n = 61). Correlations between two variables were assessed by the Spearman's rank correlation test. (D) Correlation of CD81 concentration in fraction p5 given in RU/mL and serum AST level given in U/mL in all patients with chronic HCV infection, including patients with elevated (▵) and persistently normal (○) ALT levels (n = 61). Correlations between two variables were assessed by the Spearman's rank correlation test.

We next investigated whether CD81 concentration in the p5 fraction can be quantified. For this purpose, different dilutions of Huh7cell lysates were detected by Western blot analysis and quantified by densitometry. As shown in Figure 1 C and D, this approach allowed semiquantitative assessment of CD81 in the p5 fraction (CD81-p5). In the subsequent experiments, different dilutions of Huh7 lysates covering the range of serum CD81-p5 were used as reference standard. Stock solutions from the same lysate were used for all analyses (Figure 2A).

### Association of serum CD81-p5 with chronic hepatitis C virus infection

Aim of this study was to investigate whether soluble CD81 levels are increased in patients with chronic hepatitis C. Therefore, the concentration of soluble CD81 in the exosomal fraction p5 was compared between a cohort of 23 healthy controls and 37 patients with chronic hepatitis C. As shown in Figure 2, the serum CD81 concentration in fraction p5 was significantly higher in patients with chronic hepatitis C compared with healthy controls (Figure 2 A, B). In patients with chronic hepatitis C, CD81-p5 was neither associated with patients characteristics such as age and gender (Table 1). Apparently, there was no association between CD81-p5 and HCV genotype, however, reliable conclusions on the association between HCV genotype and soluble CD81 cannot be drawn due to imbalanced genotype distribution in the different cohorts. To investigate whether HCV infection is associated with an increase of serum obtained CD81, CD81-p5 was analyzed in a cohort of patients with long term cure of HCV after pegylated interferon-alfa based antiviral therapy and compared with CD81-p5 in healthy controls and in patients with chronic hepatitis C. In patients with cured hepatitis C the CD81-p5 level was similar to healthy controls and significantly lower than in patients with chronic hepatitis C (Figure 2 B).

**Table 1:** Baseline characteristics of patients and healthy controls.

		Patients with chronic HCV infection		Patients with SVR	Healthy volunteers
		normal	elevated		
		ALT	ALT		
		(n = 24)	(n = 37)	(n = 7)	(n = 23)
***Demography***					
Age (mean ± SD)		50.8±11.8	53.3±10.2	63.6±11.8	29.4±5.7
Gender (male/female)		10/14	15/22	4/3	12/11
***Biochemistry***					
ALT (U/L; mean ± SD)		41.4±12.8	145.3±108.3	28.7±10.8	26.5±4.9
AST (U/L; mean ± SD)		39.9±16.9	106.7±61.8	38.4±4.1	29.1±5.5
GGT (U/L; mean ± SD)		49.3±48.1	99.8±71.3	24.5±10.6	30.0±6.8
***Histological activity index***					
I	(median [min., max.])	1 (1, 1)	1 (1, 3)	n.d.	n.a.
II	(median [min., max.])	1 (1, 1)	1 (1, 1)	n.d.	n.a.
III	(median [min., max.])	3 (1, 3)	3 (1,3)	n.d.	n.a.
IV	(median [min., max.])	1 (1, 3)	3 (1,3)	n.d.	n.a.
***Virology***					
Genotype distribution (1/2/3)		17/4/3	32/1/4	n.d.	n.a.
HCV RNA (log IU/mL, mean ± SD)		5.8±0.6	6.1±0.6	n.d.	n.a.

ALT, alanine transaminase; AST, aspartate transaminase; GGT, gamma-glutamyl transferase (the upper limit of normal was 50 U/L, 50 U/L, 39 U/L for ALT, AST and GGT); max., maximum; min; minimum; n.a., not applicable; n.d., not done NR, nonresponse SD, standard deviation; SVR, sustained virologic response.

### CD81-p5 concentration and inflammatory activity

Exosomal CD81 may also be associated with inflammatory activity. To investigate a potential association between the CD81 concentration in the p5 fraction and liver inflammation, CD81-p5 was analyzed in a cohort of patients with chronic hepatitis C and persistently normal ALT levels. In the present study, patients with chronic hepatitis C and persistently normal ALT levels showed lower CD81-p5 levels than patients with chronic hepatitis C and elevated ALT levels and comparable levels of CD81-p5 with healthy controls and patients with cured HCV.

Overall, the CD81-p5 level was associated with serum ALT activity in patients with chronic hepatitis C with or without ALT elevation (r = 0.372, p = 0.003, Figure 2C). Furthermore, we observed a significant association between CD81-p5 and aspartate aminotransferase serum levels (r = 0.306, p = 0.017; Figure 2D). Both associations were significant for the complete cohort of patient with chronic HCV infection only, but not for subgroups with normal or elevated ALT levels, respectively (p>0.2 for both). Gamma-glutamyl transferase levels were significantly associated with ALT and AST serum levels but not with CD81-p5 (r = 0.589; p<0.001; r = 0.576, p<0.001; r = 0.228, p = 0.08, respectively).

**Figure 3:**
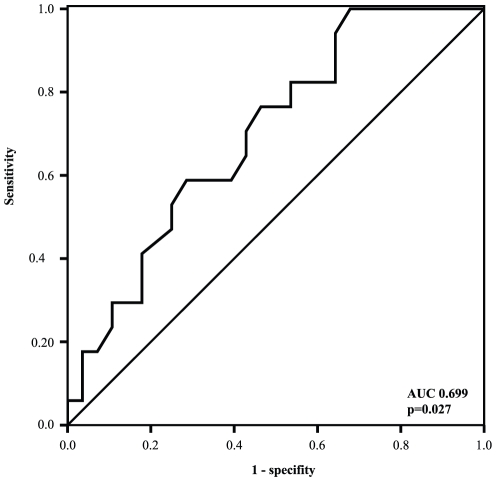
Receiver operating characteristic showing the association between soluble CD81 serum concentration and fibrosis. Higher CD81-p5 levels were associated with sever fibroses (area under curve, AUC, 0.699; p = 0.027).

We also analyzed the association between CD81-p5 and histological liver damage. CD81-p5 was significantly associated with severe vs. non severe fibrosis (area under curve 0.699, p = 0.027, Figure 3). However, no correlation was noticed between CD81-p5 and histological necroinflammatory activity (p>0.2). Serum ALT and AST levels also did not show significant correlations with necroinflammatory activity (p>0.2 for both).

### CD81-p5 and HCV RNA

**Figure 4:**
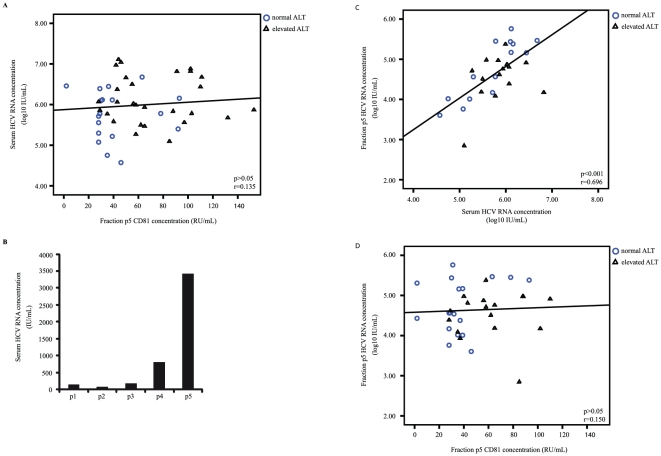
Association of HCV RNA levels and the concentration of CD81 in serum fraction p5 in patients with chronic hepatitis C and persistently normal (○) and elevated (▵) ALT levels. Correlations between two variables were assessed by the Spearman's rank correlation test. (A) Serum HCV RNA and CD81 concentration in fraction p5 were not correlated. (B) Quantification of HCV RNA in the different serum fractions showing the highest HCV RNA concentration in fraction p5. (C) Correlation between HCV RNA concentration in fraction p5 and serum showing high correlation between HCV RNA concentration in unfractionated and enriched serum. (D) Concentrations of HCV RNA in fraction p5 and CD81 fraction p5 showed no correlation. P values and correlation coefficient r are given for each correlation.

As HCV binds to CD81, the CD81 concentration in fraction p5 may be associated with the HCV RNA level. The HCV RNA concentration was quantified in unfractionated serum and in the enriched serum fraction p5 in all patients with chronic HCV infection (Figure 4 A, B). The HCV RNA concentration in unfractionated serum was significantly correlated with the HCV RNA concentration in fraction p5 (p<0.001; r = 0.696, Figure 4C). However, the CD81 concentration in fraction p5 neither correlated with the HCV RNA concentration in unfractionated serum (p = 0.373; r = 0.135, Figure 4A) nor with the HCV RNA concentration in fraction p5 (p = 0.39; r = 0.15, Figure 4D). Accordant analyses of the subgroups of patients with persistently normal or elevated ALT serum levels showed no correlation between HCV RNA concentration in serum or HCV RNA concentration in fraction p5 with CD81-p5, respectively (p>0.2 for both subgroups).

## Discussion

The tetraspanin CD81 is an essential HCV hepatocyte cell entry receptor [13], [14]. Interplay between HCV and CD81 expressed on PBMC has been related to HCV persistence as well as modulation of extrahepatic manifestations of chronic HCV infection [16]–[20], [27].

CD81 containing exosomes have not been studied in larger cohorts of patients with chronic hepatitis C, so far. Exosomes are small vesicles secreted by vital cells only, amongst others considered to modify immune response in viral and malignant diseases [23], [30]–[33]. For instance, exosome mediated infection of cells has been described in human immunodeficiency virus infection [32]. Moreover, exosome mediated intercellular transfer of CD81 and enhancement of CD81 concentration on cell surfaces by mergence has been observed [23]. Therefore, exosomal CD81 taken up by target cells, e.g. hepatocytes, could increase HCV receptor density and facilitate HCV entry, and HCV particles bound to exosomal CD81 could moreover represent an additional HCV compartment with putative relevance for the HCV infection rate. Aim of the present study was to quantify and compare CD81 in the exosomal serum fraction in patients with chronic hepatitis C compared to healthy controls and patients with cured hepatitis, and furthermore to investigate whether soluble CD81 in the exosomal serum fraction is associated with inflammatory activity in chronic hepatitis C.

Enrichment of exosomes from plasma or serum, as performed within the current study, can be performed by differential centrifugation [23], [25]. Here, CD81 is an exosomal marker protein, and the CD81 content in the exosomal fraction can be considered to be mainly of exosomal origin [23], [25]. Therefore, we measured the concentration of soluble CD81 after differential centrifugation of a standardized volume of 1 mL serum.

In the present study, soluble CD81 levels were increased in the exosomal serum fraction p5 in patients with chronic hepatitis C compared to healthy volunteers. Moreover, in patients cured from hepatitis C, CD81 levels normalized to levels found in healthy subjects. Overall these results suggest that infection with the hepatitis C virus is associated with increase of soluble CD81.

Soluble CD81 could be associated with HCV replication. Therefore, we assessed whether soluble CD81 correlates with the HCV RNA level. In the present study, however, HCV RNA serum levels were not correlated with the CD81 concentration in the exosomal serum fraction p5. HCV RNA may also bind to exosomal CD81 which is suggested by data from Masciopinto et al., who have shown an association of HCV envelope proteins and HCV RNA with exosomal CD81 *in vitro* [25]. In the present study, HCV RNA was enriched in the exosomal serum fraction; however, the concentration of HCV RNA in the exosomal serum fraction was not correlated with the soluble CD81 concentration. Therefore, it may be concluded that exosomal CD81 is not directly involved in HCV replication.

Recently, proinflammatory exosomes have been described to contribute to inflammation e.g. in sarcoidosis [34]. To investigate whether increased soluble CD81 levels are associated with disease activity in chronic HCV infection, we compared soluble CD81 serum levels in patients with chronic hepatitis C to a cohort of patients with HCV infection but persistently normal ALT levels as ALT is a surrogate marker for apoptosis and necroinflammation in chronic hepatitis C. It is estimated that approximately 30% of patients with HCV show normal ALT levels [35]. The reason therefore is unknown, but it was suggested that an arbitrary high upper limit of normal range may contribute to this phenomenon [36]. The long term clinical outcome is not completely known.

Patients with HCV infection and persistently normal ALT levels may show a more benign course than patients with elevated ALT levels. Nevertheless, several studies described significant histological liver lesions and increased ALT levels over time in these patients [36]–[40], indicating that this specific cohort may develop liver damage in the absence of surrogate cell death markers. In the current study, soluble CD81 levels were significantly higher in patients with hepatitis C compared to patients with HCV infection and normal ALT levels. This may indicate that increase of soluble CD81 in patients with chronic hepatitis C is mostly related with HCV associated necroinflammation.

To further investigate the observed correlation between soluble CD81 levels and necroinflammatory activity, we compared CD81 levels to ALT and AST levels. Here, a significant correlation between serum ALT as well as AST levels and soluble CD81 was observed. This may indicate an association between hepatocyte cell death and higher CD81 serum levels. The association between soluble CD81 and surrogate markers of cell death could be due to release of CD81 containing exosomes or cell detritus from hepatocytes during the necroinflammatory process. However, the correlation coefficients between soluble CD81 levels and biochemical markers of liver damage, even though statistically significant, were weak. A final conclusion that soluble CD81 levels are mostly derived from inflammatory processes in patients with hepatitis C cannot be drawn from the results of the current study.

Another explanation for the increase of soluble CD81 levels in patients with chronic hepatitis C is secretion of CD81 containing exosomes by lymphocytes. Interaction of HCV particles with CD81 expressed on PBMC as well as alteration of CD81 expression on PBMC during HCV infection is well known and of clinical importance [18], [19], [41]. Potential interaction of PBMC released CD81 exosomes with HCV particles may occur and influence hepatic inflammation in chronic hepatitis C. Here, further studies defining the source of soluble CD81 in HCV infection are necessary.

In summary, the results of the present study show that chronic HCV infection is associated with increase of soluble CD81 in the exosomal serum fraction. CD81 in the exosomal fraction in patients with chronic hepatitis C appears to be associated with inflammatory activity. This is a new finding with potential implications in the understanding of HCV persistence and HCV-associated necroinflammation. In concordance, soluble CD81 levels were associated with higher stage liver fibrosis.
